# Telemedicine in research and training: spine surgeon perspectives and practices worldwide

**DOI:** 10.1007/s00586-020-06716-w

**Published:** 2021-01-22

**Authors:** Karim Shafi, Francis Lovecchio, Grant J. Riew, Dino Samartzis, Philip K. Louie, Niccole Germscheid, Howard S. An, Jason Pui Yin Cheung, Norman Chutkan, Gary Michael Mallow, Marko H. Neva, Frank M. Phillips, Daniel M. Sciubba, Mohammad El-Sharkawi, Marcelo Valacco, Michael H. McCarthy, Melvin C. Makhni, Sravisht Iyer

**Affiliations:** 1 Department of Orthopaedic Surgery, Hospital for Special Surgery, 535 East 70th St., New York, NY 10021 USA; 2 Department of Orthopaedic Surgery, Brigham and Women’s Hospital, Harvard Medical School, Boston, MA USA; 3 Department of Orthopaedic Surgery, Rush University Medical Center, Chicago, IL USA; 4 The International Spine Research and Innovation Initiative, Rush University Medical Center, Chicago, IL USA; 5 Neuroscience Institute, Virginia Mason Medical Center, Seattle, WA USA; 6 Research Department, AO Spine International, Davos, Switzerland; 7 Department of Orthopaedics & Traumatology, The University of Hong Kong, Hong Kong SAR, China; 8 Department of Orthopaedic Surgery, University of Arizona College of Medicine, Phoenix, AZ USA; 9 Department of Orthopaedic and Trauma Surgery, Tampere University Hospital, Tampere, Finland; 10 Department of Neurosurgery, John Hopkins University, Baltimore, MD USA; 11 Department of Orthopaedic and Trauma Surgery, Assiut University Medical School, Assiut, Egypt; 12 Department of Orthopaedics, Churruca Hospital de Buenos Aires, Buenos Aires, Argentina; 13 Indiana Spine Group, Carmel, IN USA

**Keywords:** Telemedicine, Spine, Research, Training, Survey

## Abstract

**Purpose:**

To utilize a global survey to elucidate spine surgeons’ perspectives towards research and resident education within telemedicine.

**Methods:**

A cross-sectional, anonymous email survey was circulated to the members of AO Spine, an international organization consisting of spine surgeons from around the world. Questions were selected and revised using a Delphi approach. A major portion of the final survey queried participants on experiences with telemedicine in training, the utility of telemedicine for research, and the efficacy of telemedicine as a teaching tool. Responses were compared by region.

**Results:**

A total of 485 surgeons completed the survey between May 15, 2020 and May 31, 2020. Though most work regularly with trainees (83.3%) and 81.8% agreed that telemedicine should be incorporated into clinical education, 61.7% of respondents stated that trainees are not present during telemedicine visits. With regards to the types of clinical education that telemedicine could provide, only 33.9% of respondents agreed that interpretation of physical exam maneuvers can be taught (mean score = − 0.28, SD =  ± 1.13). The most frequent research tasks performed over telehealth were follow-up of imaging (28.7%) and study group meetings (26.6%). Of all survey responses provided by members, there were no regional differences (*p* > 0.05 for all comparisons).

**Conclusions:**

Our study of spine surgeons worldwide noted high agreement among specialists for the implantation of telemedicine in trainee curricula, underscoring the global acceptance of this medium for patient management going forward. A greater emphasis towards trainee participation as well as establishing best practices in telemedicine are essential to equip future spine specialists with the necessary skills for navigating this emerging platform.

**Supplementary Information:**

The online version contains supplementary material available at (10.1007/s00586-020-06716-w).

## Introduction

Telemedicine, the remote delivery of clinical care through videoconferencing or audio platforms, has experienced explosive growth in light of the COVID-19 pandemic [1, 2]. Social distancing practices have drastically altered the practice of orthopaedic care, requiring the cessation of nonurgent surgeries and a need for alternative means of care delivery to high-risk populations. Providers and patients alike have rapidly turned to telemedicine as a surrogate means of establishing care [3, 4]. While pre-pandemic predictions valued the 2025 telemedicine market beyond $64 billion, the present climate suggests even further growth as pandemic precautions and social guidelines persist [5].

Early evidence has demonstrated overall provider satisfaction with telemedicine across all specialties [6–9]. Among arthroplasty and sports medicine surgeons, telemedicine has demonstrated initial utility in providing follow-up care and at-home rehabilitation to patients [10–13]. However, there remains a lack of best practices for implementing this platform into both orthopaedic surgery trainee curricula and research. As institutions convert to a “new normal” of practice management, it is imperative to consider how budding spine surgeons are best trained in a learning environment that is rapidly adapting to external forces in order to continue to deliver high-quality care.

To our knowledge, no prior studies have evaluated if or how telemedicine should be incorporated into training spine surgery residents or fellows. Given the recent ubiquity of telemedicine use and its early benefit in supporting resident training, those formulating Accreditation Council for Graduate Medical Education (ACGME) compliant curricula must consider current implementation of and attitudes towards telemedicine [14, 15]. The purpose of this study was to survey spine surgeons’ perspectives towards research and resident education within telemedicine.

## Methods

### Survey design

An anonymous, cross-sectional global survey, entitled “*Telemedicine & the Spine Surgeon: Perspectives and Practices Worldwide*”, was conducted to assess spine surgeons’ attitudes towards telemedicine (“Appendix 1”). The survey was formulated through a modified Delphi approach, in which all questions underwent four consecutive rounds of review by the multi-disciplinary study authors [16]. To maintain anonymity, no identifying information was collected from survey respondents. The final version consisted of 42 questions, covering seven critical categories as determined by the study authors.

The seventh critical category of our survey consisted of questions pertaining to telemedicine in training and research. In addition to its impact on patient care, the COVID-19 pandemic has upended how the next generation of surgeons is trained. Furthermore, it has forced providers to quickly adopt a new form of patient evaluation into their practice, raising questions on whether trainees should be formally educated on the use of telemedicine. Survey participants were asked about the presence of trainees in telemedicine visits, their stances on formal telemedicine education, and whether telemedicine may be used to teach clinical skills (i.e. physical exam, interpretation of imaging, etc.). Similarly, participants’ use of telemedicine for research activities was explored (“Appendix 1”).

### Respondent sample

The above survey was distributed via electronic mail to members of AO Spine from all 5 AO regions who elected to receive relevant surveys [1]. AO Spine is currently the largest international organization dedicated to practice and study of spine surgery, comprised of over 6000 actively practicing spine surgeons (www.aospine.org). The survey period began on May 15, 2020. An email reminder was sent on May 24, 2020, prior to the survey closing on May 31, 2020. Additionally, each AO Spine Regional Director and Regional Research Officer was approached to personally connect and reach out to (the same) members in their region (i.e. Country Council Chairs, Fellowship Officers, etc.) regarding participation.

### Statistical analyses

All questions presented were optional, thus both incomplete and completed surveys were counted in the final analysis. Incomplete data points were excluded per analysis. Given the extent of content accumulated from the 42-question survey, statistical analyses were divided into four major themes: global perspectives, challenges and benefits, telemedicine evaluation, and training and research. The present analysis is focused specifically on questions pertaining to the utility of telemedicine in training and research. Survey respondents were grouped into five geographical regions, namely Africa, Asia-Pacific, Europe, North America, and South America. Participants were stratified by geographical region, and responses were subsequently compared. Categorical variables were analyzed using Pearson’s chi-square test, and continuous variables were assessed using Mann–Whitney U tests or ANOVA as appropriate. All statistical analyses were performed using SPSS Version 25.0 (IBM, Armonk, NY). Statistical significance was set at *p* < 0.05.

## Results

### Demographics

A total of 485 surgeons completed the survey between May 15, 2020 and May 31, 2020. The majority of the sample included both orthopaedic spine surgeons (68.5%, *n* = 332) and neurosurgeons (29.7%, *n* = 144). The majority of respondents belonged to either an Academic/University hospital department (34%, *n* = 164) or a combined Academic/Private practice (26.6%, *n* = 128). 85.4% of surgeons (*n* = 408) stated that they practice in an urban setting, while the remainder work in a suburban (13.2%, *n* = 63) or rural community (1.5%, *n* = 7) (Table 1). While responses to survey questions regarding the use of telemedicine in education and research were optional, there were over 200 responses provided to each of these questions (Tables 2, 3 and 4).

**Table 1:** Demographic data

	*n*^a^	Percentage^b^ (%)		*n*^a^	Percentage^b^ (%)
*Sex*			*Specialty*		
Male	446	94.5	Orthopaedics	332	68.5
Female	26	5.5	Neurosurgery	144	29.7
*Age (years)*			Trauma	50	10.3
25–34	56	11.7	Pediatric surgery	16	3.3
35–44	173	36.1	Other	14	2.9
45–54	160	33.4	*Practice type*		
55–64	73	15.2	Academic/University Hospital	164	34.0
65+	17	3.5	"Privademic" (academic/Private combined)	128	26.6
*Geographic region*			Private group, < 10 practitioners	58	12.0
Africa	95	19.9	Private group, > 10 practitioners	20	4.1
Asia Pacific	94	19.7	Individual practice	35	7.3
Europe	116	24.3	Government/Military Hospital	34	7.1
North America	45	9.4	Hospital employee	29	6.0
South America	127	26.6	Other	14	2.9
*Estimated population hospital serves*			*Hospital community*		
< 100,000	46	9.6	Urban	408	85.4
100,000–500,000	118	24.7	Suburban	63	13.2
500,000–1,000,000	100	21.0	Rural	7	1.5
1,000,000–2,000,000	67	14.0	Total respondents	485	100.0
> 2,000,000	146	30.6			

^a^Number of respondents/votes

^b^Percentages were calculated based on total responses per question, not total number of survey responses

**Table 2:** Telemedicine and training

	*N*	% respondents
*Are trainees (residents, fellows, *etc*.) present during your telemedicine visits with patients?*		
Yes	48	21.6
No	137	61.7
I do not normally work with trainees	37	16.7
Total	222	
*Are other doctors present during your telemedicine visits with patients?*		
Yes; other spine surgeons	17	7.7
Yes; other surgeons (not spine, i.e. approach surgeons	10	4.5
Yes; the patient's primary care provider	11	5
No	183	82.8
Total	221	
*Do you think telemedicine should be part of a residency/fellowship candidate's training curriculum in the clinical setting?*		
Yes	180	81.8
No	40	18.2
Total	220

**Table 3:** Telemedicine and training likert values

Question	Strongly disagree (− 2) (%)	Disagree (− 1) (%)	Neutral (0) (%)	Agree (1) (%)	Strongly agree (2) (%)	Mean likert score	SD	Total responses
Telemedicine should be a part of the medical school curriculum	3.70	4.10	18.30	59.60	14.20	0.766	0.88	218
Telemedicine should be incorporated as part of residency/fellowship training	3.20	4.60	20.20	56.00	16.10	0.771	0.89	218
Directed history taking can be taught via telemedicine	1.40	5.00	16.10	62.80	14.70	0.844	0.78	218
Interpretation of physical exam maneuvers can be taught via telemedicine	11.50	24.80	24.30	33.90	5.50	− 0.28	1.13	218
Interpretation of imaging studies can be taught via telemedicine	1.40	4.60	13.30	56.90	23.90	0.973	0.83	218
I prefer teaching via telemedicine to in person	16.10	30.30	34.90	16.10	2.80	− 0.408	1.03	218
Teaching over telemedicine is as effective as in-person teaching	12.00	38.20	28.10	16.60	5.10	− 0.355	1.03	218

Respondents request to respond as follows: − 2 (strongly disagree), − 1 (disagree), 0 (neutral), 1 (agree), 2 (strongly agree)

**Table 4:** Telemedicine and training likert values

Which of the following research activities are you performing over telemedicine?		
	*N*	% Total
Patient recruitment/enrolment (obtaining patient consent)	75	15.50
Follow up physical examination	52	10.70
Follow up Health-related Quality of life (HRQL) & other survey questionnaires	107	22.10
Follow up radiographs/imaging	139	28.70
Discuss research findings with research participants	89	18.40
Study group meetings	129	26.60
Other	15	3.10

### Telemedicine and training

With regards to provider presence, 21.6% of survey respondents (*n* = 48) noted that trainees are present during their telemedicine visits, while the majority of respondents stated that no trainees are present (61.7% of respondents, *n* = 137). 16.7% of respondents stated that they do not normally work with trainees (*n* = 37). Similarly, 82.8% of surgeons (*n* = 183 respondents) noted that other providers, including other spine surgeons, approach surgeons, or primary care providers, are not present during telemedicine visits. When asked if telemedicine should be incorporated into resident/fellow training in the clinical setting, 81.8% stated “Yes” (*n* = 180) (Table 2).

A total of 218 respondents completed questions related to telemedicine and training. Mean Likert scale values demonstrated that more than half of respondents agreed with the statements that telemedicine should be part of the medical school curriculum (mean score = 0.77, SD = ± 0.88), and that telemedicine should be incorporated as part of residency/fellowship training (mean score = 0.77, SD = ± 0.89). Additionally, most respondents agreed that both directed history taking (mean score = 0.84, SD = ± 0.78) and interpretation of imaging studies (mean score = 0.97, SD =  ± 0.83) can be taught via telemedicine. Only 33.9% of respondents agreed that interpretation of physical exam maneuvers can be taught via telemedicine (mean score = − 0.28, SD = ± 1.13), and most respondents disagreed with the statements “*I prefer teaching *via* telemedicine to in-person”* (mean score = −0.41, SD = ± 1.03) and “*teaching over telemedicine is as effective as in-person teaching*” (mean score = − 0.36, SD = ± 1.03) (Table 3).

### Telemedicine and research

AO Spine members were also surveyed with regards to the use of telemedicine and research. 28.7% of surgeons stated that they have utilized telemedicine for follow-up of imaging (*n* = 139). Other more frequently cited uses of telemedicine were study group meetings (26.6%, *n* = 129 and for follow-up of Health-related Quality of Life (HRQL) or other survey questionnaires (22.1%, *n* = 107) (Table 4).

### Comparison by region and age

Of the survey responses provided by members, there was no statistically significant difference when stratified by region and by age (> 55 years old) (Pearson Chi-squared > 0.05 for all associations) (“Appendices 2, 3, 4”).

## Discussion

The present study focused on AO Spine members’ contemporary perspectives towards telemedicine with regards to training and research. Most practitioners (61.7%, *n* = 137) reported that they currently do not have trainees present during telemedicine visits. Nevertheless, 81.8% of respondents stated that they believe telemedicine should be incorporated into resident and/or fellowship training in a clinical setting (Table 2). Provider attitudes towards telemedicine and training were somewhat mixed; mean Likert scores were in highest agreement for the utility of telemedicine for teaching history-taking, evaluating imaging, and the incorporation of telemedicine into training curricula. The lowest mean Likert score was reported for the interpretation of physical findings (mean score = − 0.28, SD = ± 1.13). With regards to research, surveyed members most commonly utilize telemedicine for follow-up of radiographs/imaging or study group meetings (Table 4). Our findings suggested that respondents’ perspective did not differ by geographic region (Pearson Chi-squared > 0.05 for all scores).

As telemedicine becomes increasingly utilized in musculoskeletal care and appears to be a prominent platform moving forward, it is critical that budding spine surgeons undergo sufficient training with virtual healthcare platforms in order to deliver high-quality care. Our results suggest a gap between the current exposure trainees have to what spine surgeons feel trainees *should have* in practice. Traditionally, junior and attending physicians both perform independent evaluations of a patient, followed by a debriefing. Here, trainees may receive valuable feedback with regards to their interviewing, examination and diagnostic skills, and bedside manner [17]. This process is also crucial for training in telemedicine. Afshari et al. [18] trialled a didactic and clinical-based virtual healthcare curriculum for neurology residents, formulated to assess trainees attitudes toward and challenges faced when utilizing telemedicine. When evaluating subjects’ performance, the authors found that residents adapted quickly to videoconferencing systems but felt less comfortable in building a strong physician–patient relationship in comparison with an in-person visit. Similar literature has suggested that developing a strong “webside manner” requires sufficient time and practice [19, 20]. Moving forward, we feel that senior spine surgeons should be encouraged to involve their peers and juniors when performing telemedicine visits, allowing for more frequent and effective exposure.

Fortunately, implementation of formal telemedicine training into trainee curricula has already been initiated. In 2016, the American Medical Association implemented a policy “encouraging the accrediting bodies for both undergraduate and graduate medical education to include core competencies for telemedicine in their programs.”[21] Our survey results support these changes; the majority of respondents either agreed or strongly agreed that telemedicine should both be part of the medical school curriculum and incorporated as part of residency/fellowship training (Table 3). Jagolino et al. [22] incorporated a formal telemedicine rotation into their neurology fellowship program. From their early experiences, the authors reported that fellows’ attitudes towards telemedicine were largely positive, and that 81% of subjects agreed that telemedicine should be required in a one-year ACGME-accredited vascular fellowship. Similar pilot curricula have been developed within medical schools, internal medicine, family medicine, and dermatology residencies, with modest early success [14, 23–25].

Despite these early advances, implementation of formal telemedicine training is challenged by a diverse array of platforms and technologies and their associated technical difficulties and the lack of standardized physical examination practices [14, 26, 27]. Our results indicate that, while spine surgeons may be as comfortable imparting history-taking skills and evaluation of imaging via telemedicine, the inherent remote nature of the visit creates a unique challenge in teaching orthopaedic/neurologic physical examination maneuvers (Table 3). While surrogate physical exam maneuvers have been suggested [27, 28], we recognize that this aspect of training may be best learned in a traditional manner. Moving forward, establishing a uniform spine telemedicine visit structure, with standardization of all clinical components of the visit, will likely enhance the trainee experience.

Few prior studies have evaluated the utility of telemedicine when conducting musculoskeletal research. While telemedicine has provided a surrogate infrastructure to traditional, in-person clinical care during the COVID-19 crisis, our survey suggests that musculoskeletal research has not yet benefitted from the gross introduction of videoconferencing as seen in the clinical setting. 28.7% (*n* = 139) and 26.6% (*n* = 129) of respondents noted that they have utilized telemedicine for follow-up of imaging and/or study group meetings. Similarly, Upadhaya et al. [29] noted that patient enrolment in active oncology clinical trials have been severely negatively affected, including over 200 interventional trials between March and April of 2020. Cancer researchers have noted that temporary regulatory waivers by the U.S. Food and Drug Administration (FDA) may make telemedicine a useful tool in maintaining and furthering medical research during this pandemic [29–31]. The cost and convenience of telemedicine, coupled with permissive use of previously non-HIPAA complaint videoconferencing platforms (eg. Zoom, Skype for Business, GoogleChat) [32], may provide spine surgeons with a key tool in conducting research moving forward.

Our investigation was not without limitations. First, responses to survey questions were optional; therefore, our results fail to capture the current practices of all respondents (*n* = 485). In addition, certain regions were likely underrepresented (e.g. North America). Second, while the majority of respondents were orthopaedic surgeons, neurosurgeons, traumatologists, pediatric surgeons, and other practicing physicians also contributed. The diversity of pathologies and patients that these practitioners treat, coupled with the potential for varying levels of experience, likely create similarly unique telemedicine practices. However, we do not suspect that this would significantly influence their perspectives towards research and education within telemedicine. Third, we note that our lack of statistical power limits our ability for stratification and comparison by region. Fourth, we did not compare attitudes towards telehealth across different practice types, hospital communities or population size served. Lastly, as telemedicine was adopted extremely rapidly, negative attitudes towards telemedicine may be rooted in inexperience. As practitioners and trainees become more comfortable with remote healthcare, the potential benefits of telemedicine as a clinical and training tool may become more evident.

## Conclusions

Our study of spine surgeons worldwide noted high agreement among specialists for the implantation of telemedicine in trainee curricula. Over 80% of members responding agreed that telemedicine deserves a formal role in the clinical setting in residencies and fellowship programs. However, surgeons disagreed that telemedicine is as effective as in-person training or is a preferred method of teaching. Survey respondent perspectives towards the utility of training and research within telemedicine did not differ based on geographical region, which underscores the global acceptance and universal appeal of this medium for patient management. Given the rapid surge in telemedicine use, we believe that assessing contemporary perspectives with regards to telemedicine and research and training is key to improving virtual healthcare quality. While significant progress has been made in addressing the technical challenges and unfamiliarity associated with telemedicine, further attention must be paid towards establishing best practices in telemedicine training and research, in order to fully equip future spine specialists with the necessary skills for navigating this novel platform.

## Supplementary Information

Supplementary material 1 (DOCX 42 kb)

## References

[1] Louie PK, Harada GK, McCarthy MH (2020). The impact of COVID-19 pandemic on spine surgeons worldwide. Glob Spine J.

[2] Swiatek P, Weiner J, Johnson D et al (2021) COVID-19 and the rise of virtual medicine in spine surgery: a worldwide study 10.1007/s00586-020-06714-y PMC7811348 33452925

[3] Miliard M (2020) Telehealth set for “tsunami of growth,” says Frost & Sullivan. In: Healthc. IT News

[4] Portnoy J, Waller M, Elliott T (2020). Telemedicine in the era of COVID-19. J Allergy Clin Immunol Pract..

[5] U.S. Telemedicine Market Statistics 2019–2025 (2020) Share forecasts, trends & growth drivers. MarketWatch

[6] Kwok J, Olayiwola JN, Knox M (2018). Electronic consultation system demonstrates educational benefit for primary care providers. J Telemed Telecare.

[7] Lee MS, Ray KN, Mehrotra A (2018). Primary care practitioners’ perceptions of electronic consult systems: a qualitative analysis. JAMA Int Med.

[8] Good DW, Lui DF, Leonard M (2012). Skype: a tool for functional assessment in orthopaedic research. J. Telemed. Telecare.

[9] Lamminen H, Nevalainen J, Alho A (1996). Experimental telemedicine in orthopaedics. J Telemed Telecare.

[10] Buvik A, Bugge E, Knutsen G (2016). Quality of care for remote orthopaedic consultations using telemedicine: a randomised controlled trial. BMC Health Serv Res.

[11] Kane LT, Thakar O, Jamgochian G (2020). The role of telehealth as a platform for postoperative visits following rotator cuff repair: a prospective, randomized controlled trial. J Shoulder Elb Surg.

[12] Nicholson KJ, Millhouse PW, Pflug E (2018). Cervical sagittal range of motion as a predictor of symptom severity in cervical spondylotic myelopathy. Spine.

[13] Sharareh B, Schwarzkopf R (2014). Effectiveness of telemedical applications in postoperative follow-up after total joint arthroplasty. J Arthroplasty.

[14] Lee MS, Nambudiri V (2019). Integrating telemedicine into training: adding value to graduate medical education through electronic consultations. J Grad Med Educ.

[15] ACGME (2020) ACGME Common Program Requirements (Residency). Accredit Counc Med Educ

[16] Okoli C, Pawlowski SD (2004). The Delphi method as a research tool: an example, design considerations and applications. Inf Manag.

[17] Clark R, Evans EB, Ivey FM (1989). Characteristics of successful and unsuccessful applicants to orthopedic residency training programs. Clin Orthop Relat Res.

[18] Afshari M, Witek NP, Galifianakis NB (2019). Education Research: an experiential outpatient teleneurology curriculum for residents. Neurology.

[19] McConnochie KM (2019). Webside manner: a key to high-quality primary care telemedicine for all. Telemed. e-Health.

[20] Chua IS, Jackson V, Kamdar M (2020). Webside manner during the COVID-19 pandemic: maintaining human connection during virtual visits. J Palliat Med.

[21] AMA (2016) AMA encourages telemedicine training for medical students, residents

[22] Jagolino AL, Jia J, Gildersleeve K (2016). A call for formal telemedicine training during stroke fellowship. Neurology.

[23] Wanat KA, Newman S, Finney KM (2016). Teledermatology education: current use of teledermatology in US residency programs. J. Grad. Med. Educ.

[24] Williams CM, Kedar I, Smith L (2005). Teledermatology education for internal medicine residents. J Am Acad Dermatol.

[25] Kamdar N (2019) It’s time for ACGME to require telemedicine training for residents. Op-Med

[26] Wongworawat MD, Capistrant G, Stephenson JM (2017). The opportunity awaits to lead orthopaedic telehealth innovation. J Bone Jt Surg Am.

[27] Tanaka MJ, Oh LS, Martin SD, Berkson EM (2020). Telemedicine in the era of COVID-19: the virtual orthopaedic examination. J Bone Jt Surg Am.

[28] Iyer S, Shafi K, Lovecchio F (2020). The spine physical examination using telemedicine: strategies and best practices. Glob spine J.

[29] Upadhaya S, Yu JX, Oliva C (2020). Impact of COVID-19 on oncology clinical trials. Nat Rev Drug Discov.

[30] Waterhouse DM, Harvey RD, Hurley P (2020). Early impact of COVID-19 on the conduct of oncology clinical trials and long-term opportunities for transformation: findings from an American society of clinical oncology survey. JCO Oncol Pract.

[31] Kenneth Y, Allison Fulton MG (2020) Going virtual: clinical trials, telemedicine, electronic medical records, and all thatle. SheppardMullin

[32] U.S. Department of Health and Human Services (2020) Notification of enforcement discretion for telehealth remote communications during the COVID-19 nationwide public health emergency. HHS gov

