# Supplementary material for: Telemedicine in research and training: spine surgeon perspectives and practices worldwide
Source: Eur Spine J. 2021 Jan 22;30(8):2143–9. doi: 10.1007/s00586-020-06716-w (PMC7820826; doi:10.1007/s00586-020-06716-w)
Supplement: Supplementary file 1 — Supplementary material 1 (DOCX 42 kb) [file 586_2020_6716_MOESM1_ESM.docx]

**Appendix 1. Survey**

**Telemedicine & the Spine Surgeon – Spine Surgeon Perspectives and Practices Worldwide**

**Purpose:** Given the need for social distancing with COVID-19, telemedicine services have expanded around the world. We define telemedicine in this survey as clinical care provided remotely through videoconferencing or telephone. This survey seeks to determine:

1. The extent of spine surgeon adoption of telemedicine
2. Satisfaction with telemedicine
3. Comparison of telemedicine to in-person visits
4. The use of telemedicine in research and training
5. Variations in perspectives and practices worldwide

The survey will not take more than **5-10 minutes** to complete.

Information obtained from this survey will be kept strictly confidential. The identity of all survey participants will remain anonymous. The findings of this survey will be disseminated via social media, journals, and other media platforms.

Deadline to respond is **May 31, 2020**.

Thank you in advance for taking the time to fill out this survey.

**Demographic Data**

1. Country of Spine Practice (**select one**)
2. Region of Spine Practice

a) Africa

b) Asia

c) Australia

d) Europe

f) North America

g) South America

1. What is your gender?
   1. Male
   2. Female
2. Age
   1. 25-34
   2. 35-44
   3. 45-54
   4. 55-64
   5. 65 or older
3. How many years have you been practicing spine surgery?
   1. <5
   2. 5-10
   3. 10-15
   4. 15-20
   5. 20+
4. What is your specialty?
   1. Orthopedics
   2. Neurosurgery
   3. Trauma
   4. Pediatric Surgery
   5. Other (please specify)
5. What percent (%) of your practice is (sum total should be 100%):
   1. Adult Deformity
   2. Degenerative
   3. Pediatric
   4. Trauma/Spinal Cord Injury
   5. Tumor
   6. Infections
6. Practice Type
   1. Academic/University affiliated
   2. “Privademic” (Academic/Private combined)
   3. Private group, < 10 practitioners
   4. Private group, > 10 practitioners
   5. Individual practice
   6. Government/Military Hospital
   7. Hospital Employee
   8. Other (please specify)
7. How would you define your hospital community?
   1. Urban
   2. Suburban
   3. Rural
8. What is the approximate population your hospital’s serves?
   1. < 100,000
   2. 100,000 – 500,000
   3. 500,000 – 1,000,000
   4. 1,000,000 – 2,000,000
   5. > 2,000,000
9. Approximately what percentage of your time at work do you devote to the following:
   1. Research (Slider from 0 – 100%)
   2. Clinical Care (Slider from 0 – 100%)
   3. Teaching (Slider from 0 – 100%)

**General thoughts on Telemedicine**

1. Over the past few weeks, have you seen patients via telemedicine?
   1. Yes
   2. No, we do not have telemedicine options available, but I am a believer in telemedicine
   3. No, I am not using telemedicine. I do not think telemedicine give any advantage
2. Over the past few weeks, what percentage of your patients have you seen via telemedicine?
   1. Percentage of patients: (Slider from 0 – 100%)
3. What is your main type of telemedicine platform that you use?
   1. Secure EMR-Integrated System
   2. Secure non-EMR-Integrated System
   3. Non-secure (Facetime, Skype, etc.)
   4. Phone Call (no video)
   5. Other (please specify)
   6. None
4. Enter the name of the type of telemedicine platform you used
   1. I don't know
   2. System/Platform Name: ___________________
5. The telemedicine platform was easy to use.
   1. Strongly agree
   2. Agree
   3. Undecided
   4. Disagree
   5. Strongly Disagree
6. How many telemedicine visits have you done?

a) <10

b) 11-25

c) 26-50

d) 50-100

e) 100+

1. How has your opinion about telemedicine changed as you have done more visits?

a) better than it was

b) worse than it was

c) my opinion has not changed

Comments:

1. How often do **technical difficulties delay or disrupt** the visit?
   1. Often (50%+)
   2. Frequently (30-50%)
   3. Sometimes (15-30%)
   4. Rarely (0-15%)
   5. Never (0%)
2. A telemedicine visit requires ____ of my time than an in-person visit
   1. More
   2. The same amount
   3. Less
3. Have ***you*** experienced any of the following challenges when delivering care via telemedicine? (select ***all that apply***)
   1. Lack of access to internet
   2. Lack of access to computer/phone with camera
   3. Lack of access to telephone
   4. Lack of technological literacy needed to use telemedicine
   5. Technology implementation and maintenance costs
   6. Decreased ability to perform physical examinations
   7. Possible increased medicolegal exposure
   8. Lack of reimbursement parity compared to traditional visits
   9. Unclear billing codes
   10. Regulatory barriers
   11. Other (please specify)
4. Have **your patients** experienced any of the following challenges when receiving care via telemedicine? (select all that apply)
   1. Lack of patient access to computer/phone with camera
   2. Lack of patient access to telephone
   3. Lack of technological literacy needed to use telemedicine
   4. Perceived lack of privacy
   5. Concern over paying for care received over telemedicine
   6. Other (please specify)
5. For **your patients**, how do you perceive the following statements:
   -2 (strongly disagree) ------ -1 (disagree) ------ 0 (neutral) ------- 1 (agree) ------ 2 (strongly agree)
   1. The patients I see via telemedicine are generally younger
   2. Older patients have a difficult time using telemedicine platforms
   3. Telemedicine options are not available to patients with lower income levels
   4. Patients seen over telemedicine tend to have a higher level of education
   5. Patients seen over telemedicine are less likely to be minorities
   6. Telemedicine is not significantly impacted by patient demographic factors
6. For y**our practice,** how do you perceive the following statements?

-2 (strongly disagree) ------ -1 (disagree) ------ 0 (neutral) ------- 1 (agree) ------ 2 (strongly agree)

- 1. Telemedicine increases patient satisfaction
  2. Telemedicine increases patient convenience
  3. Telemedicine increases provider convenience
  4. Telemedicine increases access to care for rural/international patients (patients from long distance)
  5. Telemedicine **decreases** overhead for providers
  6. Telemedicine **decreases** societal cost-savings (travel expenses, decreased hospital transfers, etc)

1. **Before COVID-19** and social distancing mandates, what percentage of your practice were telemedicine visits? (Rate each answer between 0-100%)
   1. New patient
   2. Follow-up visits before surgery (eg, check-in after injection, review of new imaging, etc.)
   3. Postoperative visits
2. **During COVID-19** and social distancing mandates, what percentage of your practice have been telemedicine visits? (Rate each answer between 0-100%)
   1. New patient
   2. Follow-up visits before surgery (eg, check-in after injection, review of new imaging, etc.)
   3. Postoperative visits
3. If you or a family member were a patient, how do you perceive the following statements:

-2 (strongly disagree) ------ -1 (disagree) ------ 0 (neutral) ------- 1 (agree) ------ 2 (strongly agree)

I believe:

1. The initial appointment can be done through telemedicine
2. Imaging review can be done over telemedicine
3. Post-operative care can be done through telemedicine
4. Patients should be seen at least once in person before being scheduled for surgery
5. Patients should be seen at least once in person postoperatively
6. It is clear to me how to charge for telemedicine

**Comparison of Telemedicine to In-person Visits**

How does telemedicine compare to in-person visits for the: ***(Presented as a grid)***

1. Ability to take a patient history
   1. telemedicine much better
   2. telemedicine slightly better
   3. Equivalent
   4. telemedicine slightly worse
   5. telemedicine much worse
2. Ability to localize pain
   1. telemedicine much better
   2. telemedicine slightly better
   3. Equivalent
   4. telemedicine slightly worse
   5. telemedicine much worse
3. Ability to assess neurologic deficits
   1. telemedicine much better
   2. telemedicine slightly better
   3. Equivalent
   4. telemedicine slightly worse
   5. telemedicine much worse
4. Ability to assess myelopathy
   1. telemedicine much better
   2. telemedicine slightly better
   3. Equivalent
   4. telemedicine slightly worse
   5. telemedicine much worse
5. Ability to assess spinal deformity
   1. telemedicine much better
   2. telemedicine slightly better
   3. Equivalent
   4. telemedicine slightly worse
   5. telemedicine much worse
6. Ability to perform provocative tests (straight leg raise, Spurling’s, Lhermitte’s)
   1. telemedicine much better
   2. telemedicine slightly better
   3. Equivalent
   4. telemedicine slightly worse
   5. telemedicine much worse
7. Ability to review imaging and explain to patients
   1. telemedicine much better
   2. telemedicine slightly better
   3. Equivalent
   4. telemedicine slightly worse
   5. telemedicine much worse
8. Ability to make an accurate diagnosis
   1. telemedicine much better
   2. telemedicine slightly better
   3. Equivalent
   4. telemedicine slightly worse
   5. telemedicine much worse
9. Ability to formulate and communicate a treatment plan
   1. telemedicine much better
   2. telemedicine slightly better
   3. Equivalent
   4. telemedicine slightly worse
   5. telemedicine much worse
10. For a telemedicine visit in which you request imaging for a patient, where does that test occur?
    1. Same location as for in-person visit
    2. Different location
    3. The patient can choose the location
11. For a telemedicine visit in which you request a laboratory test for a patient, where does that test occur?
    1. Same location as for in-person visit
    2. Different location
    3. The patient can choose the location
12. In general, how comfortable do you feel performing surgery after a telemedicine evaluation?
    1. Extremely comfortable, I do not need to see most patients prior to surgery
    2. Moderately comfortable, I request a short in-person visit prior to the day of surgery
    3. Slightly comfortable, I prefer a formal new patient visit prior to surgery
    4. Not at all comfortable, I do not indicate a patient for surgery over telemedicine

**Telemedicine in Training/Research**

1. Are trainees (residents, fellows, etc) present during your telemedicine visits with patients?
   1. Yes
      1. Approximately what percentage of your telemedicine visits are performed with a trainee present: (Slider 0-100%)
   2. No
   3. I don’t normally work with trainees
2. Are other doctors present during your telemedicine visits with patients?
   1. Yes, other spine surgeons
   2. Yes, other surgeons (not spine, i.e., approach surgeons)
   3. Yes, the patient’s primary care provider
   4. No
3. Approximately what percentage of your telemedicine visits are performed with the following personnel? (Rate each answer between 0-100%)
   1. Other spine surgeon
   2. Other surgeons (not spine, i.e., approach surgeons)
   3. Patient's primary care provider
4. Please provide your thoughts on the following questions as they relate to telemedicine and training:

-2 (strongly disagree) ------ -1 (disagree) ------ 0 (neutral) ------- 1 (agree) ------ 2 (strongly agree) ----- Not applicable (I have not used telemedicine for trainees)

- 1. Telemedicine should be part of the medical school curriculum
  2. Telemedicine should be incorporated as part of residency/fellowship training
  3. Directed history taking can be taught via telemedicine
  4. Interpretation of physical exam maneuvers can be taught via telemedicine
  5. Interpretation of imaging studies can be taught via telemedicine
  6. I prefer teaching via telemedicine to in person
  7. Teaching over telemedicine is **as effective** as in person teaching

1. Do you think telemedicine should be part of a residency/fellowship candidate's training curriculum in the clinical setting?
   1. Yes
   2. No
2. Of the time spent by residency/fellowship candidates in the clinic setting, what percentage should be over:
   1. Telemedicine: Slider (0-100%)
   2. In-person visits: Slider (0-100%)
3. Which of the following research activities are you performing over telemedicine? (select all that apply)
   1. Patient recruitment/enrollment (obtaining patient consent)
   2. Follow up physical examination
   3. Follow up Health-related quality of life (HRQOL) & other survey questionnaires
   4. Follow up radiographs/imaging
   5. Discuss research findings with research participants
   6. Study group meetings
   7. Other (please specify): _______

**Appendix 2. Telemedicine and Training Comparison by Region**

| ***Are trainees (residents, fellows, etc.) present during your telemedicine visits with patients?*** | | | | | |
| --- | --- | --- | --- | --- | --- |
| Region: | Africa | Asia Pacific | Europe | North America | South America |
| Yes | 7 | 17 | 10 | 4 | 10 |
| % within Region | 23.3% | 37.0% | 17.5% | 18.9% | 22.0% |
| No | 15 | 21 | 41 | 22 | 34 |
| % within Region | 50.0% | 45.7% | 71.9% | 68.8% | 64.2% |
| I don't normally work with trainees | 8 | 8 | 6 | 6 | 9 |
| % within Region | 26.7% | 17.4% | 10.5% | 18.8% | 17.0% |
| Total | 30 | 46 | 57 | 32 | 53 |
| *Pearson Chi-Square = 0.088* | | | | | |
|  |  |  |  |  |  |
| ***Are other doctors present during your telemedicine visits with patients?*** | | | | | |
| Region: | Africa | Asia Pacific | Europe | North America | South America |
| Yes, other spine surgeons | 1 | 5 | 6 | 1 | 3 |
| % within Region | 3.3% | 11.1% | 10.5% | 3.1% | 5.7% |
| Yes, other surgeons (not spine, i.e approach surgeons) | 2 | 3 | 2 | 0 | 3 |
| % within Region | 6.7% | 6.7% | 3.5% | 0.0% | 5.7% |
| Yes, patient's primary care provider | 4 | 4 | 0 | 0 | 3 |
| % within Region | 13.3% | 8.9% | 0.0% | 0.0% | 5.7% |
| No | 23 | 33 | 49 | 31 | 44 |
| % within Region | 76.7% | 73.3% | 86.0% | 96.9% | 83.0% |
| Total | 30 | 45 | 57 | 32 | 53 |
| *Pearson Chi-Square = 0.147* | | | | | |
|  |  |  |  |  |  |
| ***Do you think telemedicine should be part of a residency/fellowship candidate's training curriculum in the clinical setting?*** | | | | | |
| Region: | Africa | Asia Pacific | Europe | North America | South America |
| Yes | 26 | 39 | 43 | 28 | 42 |
| % within Region | 86.7% | 88.6% | 75.4% | 87.5% | 79.2% |
| No | 4 | 5 | 14 | 4 | 11 |
| % within Region | 13.3% | 11.4% | 24.6% | 12.5% | 20.8% |
| Total | 30 | 44 | 57 | 32 | 53 |
| *Pearson Chi-Square = 0.355* | | | | | |

**Appendix 3. Telemedicine and Training Likert Values Comparison by Region**

| ***Telemedicine should be part of the medical school curriculum*** | | | |  |  |
| --- | --- | --- | --- | --- | --- |
| Region | Africa | Asia Pacific | Europe | North America | South America |
| Strongly disagree | 0 | 1 | 3 | 1 | 3 |
| % within Region | 0.0% | 2.3% | 5.4% | 3.1% | 5.7% |
| Disagree | 1 | 3 | 1 | 1 | 3 |
| % within Region | 3.3% | 6.8% | 1.8% | 3.1% | 5.7% |
| Neutral | 5 | 5 | 17 | 6 | 7 |
| % within Region | 16.7% | 11.4% | 30.4% | 18.8% | 13.2% |
| Agree | 18 | 28 | 31 | 19 | 31 |
| % within Region | 60.0% | 63.6% | 55.4% | 59.4% | 58.5% |
| Strongly agree | 6 | 7 | 4 | 5 | 9 |
| % within Region | 20.0% | 15.9% | 7.1% | 15.6% | 17.0% |
| Total | 30 | 44 | 56 | 32 | 53 |
| *Pearson Chi-Square = 0.608* | | | | | |
|  |  |  |  |  |  |
| ***Telemedicine should be incorporated as part of residency/fellowship training*** | | | | |  |
| Region | Africa | Asia Pacific | Europe | North America | South America |
| Strongly disagree | 0 | 1 | 3 | 1 | 2 |
| % within Region | 0.0% | 2.3% | 5.4% | 3.1% | 3.8% |
| Disagree | 1 | 2 | 3 | 0 | 4 |
| % within Region | 3.3% | 4.5% | 5.4% | 0.0% | 7.5% |
| Neutral | 8 | 4 | 11 | 10 | 10 |
| % within Region | 26.7% | 9.1% | 19.6% | 31.3% | 18.9% |
| Agree | 15 | 30 | 32 | 17 | 26 |
| % within Region | 50.0% | 68.2% | 57.1% | 53.1% | 49.1% |
| Strongly agree | 6 | 7 | 7 | 4 | 11 |
| % within Region | 20.0% | 15.9% | 12.5% | 12.5% | 20.8% |
| Total | 30 | 44 | 56 | 32 | 53 |
| *Pearson Chi-Square = 0.640* | | | | | |
|  |  |  |  |  |  |
| ***Directed history-taking can be taught via telemedicine*** | | | |  |  |
| Region | Africa | Asia Pacific | Europe | North America | South America |
| Strongly disagree | 0 | 0 | 1 | 1 | 1 |
| % within Region | 0.0% | 0.0% | 1.8% | 3.1% | 1.9% |
| Disagree | 2 | 3 | 4 | 1 | 1 |
| % within Region | 6.7% | 6.8% | 7.1% | 3.1% | 1.9% |
| Neutral | 3 | 6 | 13 | 3 | 10 |
| % within Region | 10.0% | 13.6% | 23.2% | 9.4% | 18.9% |
| Agree | 21 | 29 | 32 | 19 | 33 |
| % within Region | 70.0% | 65.9% | 57.1% | 59.4% | 62.3% |
| Strongly agree | 4 | 6 | 6 | 8 | 8 |
| % within Region | 13.3% | 13.6% | 10.7% | 25.0% | 15.1% |
| Total | 30 | 44 | 56 | 32 | 53 |
| *Pearson Chi-Square = 0.786* | | | | | |
|  |  |  |  |  |  |
| ***Interpretation of physical exam maneuvers can be taught via telemedicine*** | | | | |  |
| Region | Africa | Asia Pacific | Europe | North America | South America |
| Strongly disagree | 1 | 3 | 10 | 3 | 8 |
| % within Region | 3.3% | 6.8% | 17.9% | 9.4% | 15.1% |
| Disagree | 7 | 7 | 16 | 12 | 11 |
| % within Region | 23.3% | 15.9% | 28.6% | 37.5% | 20.8% |
| Neutral | 9 | 9 | 14 | 7 | 13 |
| % within Region | 30.0% | 20.5% | 25.0% | 21.9% | 24.5% |
| Agree | 12 | 21 | 14 | 8 | 18 |
| % within Region | 40.0% | 47.7% | 25.0% | 25.0% | 34.0% |
| Strongly agree | 1 | 4 | 2 | 2 | 3 |
| % within Region | 3.3% | 9.1% | 3.6% | 6.3% | 5.7% |
| Total | 30 | 44 | 56 | 32 | 53 |
| *Pearson Chi-Square = 0.405* | | | | | |
|  |  |  |  |  |  |
| ***Interpretation of imaging studies can be taught via telemedicine*** | | | |  |  |
| Region | Africa | Asia Pacific | Europe | North America | South America |
| Strongly disagree | 0 | 0 | 2 | 1 | 0 |
| % within Region | 0.0% | 0.0% | 3.6% | 3.1% | 0.0% |
| Disagree | 2 | 2 | 3 | 0 | 3 |
| % within Region | 6.7% | 4.5% | 5.4% | 0.0% | 5.7% |
| Neutral | 2 | 8 | 8 | 4 | 7 |
| % within Region | 6.7% | 18.2% | 14.3% | 12.5% | 13.2% |
| Agree | 22 | 23 | 33 | 15 | 28 |
| % within Region | 73.3% | 52.3% | 58.9% | 46.9% | 52.8% |
| Strongly agree | 4 | 11 | 10 | 12 | 15 |
| % within Region | 13.3% | 25.0% | 17.9% | 37.5% | 28.3% |
| Total | 30 | 44 | 56 | 32 | 53 |
| *Pearson Chi-Square = 0.483* | | | | | |
|  |  |  |  |  |  |
| ***I prefer teaching via telemedicine to in-person teaching*** | | | |  |  |
| Region | Africa | Asia Pacific | Europe | North America | South America |
| Strongly disagree | 3 | 11 | 8 | 6 | 6 |
| % within Region | 10.0% | 25.0% | 14.3% | 18.8% | 11.3% |
| Disagree | 10 | 6 | 21 | 12 | 15 |
| % within Region | 33.3% | 13.6% | 37.5% | 37.5% | 28.3% |
| Neutral | 14 | 14 | 19 | 9 | 20 |
| % within Region | 46.7% | 31.8% | 33.9% | 28.1% | 37.7% |
| Agree | 3 | 10 | 8 | 4 | 10 |
| % within Region | 10.0% | 22.7% | 14.3% | 12.5% | 18.9% |
| Strongly agree | 0 | 3 | 0 | 1 | 2 |
| % within Region | 0.0% | 6.8% | 0.0% | 3.1% | 3.8% |
| Total | 30 | 44 | 56 | 32 | 53 |
| *Pearson Chi-Square = 0.260* | | | | | |
|  |  |  |  |  |  |
| ***Teaching over telemedicine is as effective as in-person teaching*** | | | |  |  |
| Region | Africa | Asia Pacific | Europe | North America | South America |
| Strongly disagree | 3 | 6 | 10 | 4 | 2 |
| % within Region | 10.0% | 13.6% | 18.2% | 12.5% | 3.8% |
| Disagree | 14 | 14 | 20 | 12 | 21 |
| % within Region | 46.7% | 31.8% | 36.4% | 37.5% | 39.6% |
| Neutral | 10 | 12 | 14 | 10 | 15 |
| % within Region | 33.3% | 27.3% | 25.5% | 31.3% | 28.3% |
| Agree | 3 | 7 | 10 | 5 | 11 |
| % within Region | 10.0% | 15.9% | 18.2% | 15.6% | 20.8% |
| Strongly agree | 0 | 5 | 1 | 1 | 4 |
| % within Region | 0.0% | 11.4% | 1.8% | 3.1% | 7.5% |
| Total | 30 | 44 | 55 | 32 | 53 |
| *Pearson Chi-Square = 0.523* | | | | | |

**Appendix 4. Telemedicine and Training Likert Values Comparison by Region**

| ***Which of the following research activities are you performing over telemedicine?*** | | | | | |
| --- | --- | --- | --- | --- | --- |
| Region: | Africa | Asia Pacific | Europe | North America | South America |
| *Patient recruitment/enrollment* | 7 | 14 | 22 | 8 | 24 |
| % within Region | 7.4% | 14.9% | 19.0% | 17.8% | 18.9% |
| *Pearson Chi-Square = 0.132* | | | | | |
| *Follow up physical examination* | 7 | 10 | 14 | 8 | 13 |
| % within Region | 7.4% | 10.6% | 12.1% | 17.8% | 10.2% |
| *Pearson Chi-Square = 0.457* | | | | | |
| *Follow up radiographs/imaging* | 20 | 26 | 35 | 19 | 37 |
| % within Region | 21.1% | 27.7% | 30.2% | 42.2% | 29.1% |
| *Pearson Chi-Square = 0.140* | | | | | |
| *Follow up Health-related quality of life (HRQL) & other survey questionnaires* | 13 | 22 | 35 | 10 | 26 |
| % within Region | 13.7% | 23.4% | 30.2% | 22.2% | 20.5% |
| *Pearson Chi-Square = 0.073* | | | | | |
| *Discuss research findings with research participants* | 16 | 15 | 24 | 9 | 22 |
| % within Region | 16.8% | 16.0% | 20.7% | 20.0% | 17.3% |
| *Pearson Chi-Square = 0.897* | | | | | |
| *Study group meetings* | 18 | 19 | 32 | 17 | 40 |
| % within Region | 18.9% | 20.2% | 27.6% | 37.8% | 31.5% |
| *Pearson Chi-Square = 0.053* | | | | | |
| *Other* | 3 | 3 | 4 | 3 | 2 |
| % within Region | 3.2% | 3.2% | 3.4% | 6.7% | 1.6% |
| *Pearson Chi-Square = 0.575* | | | | | |
